# A novel transcriptional repressor specifically regulates xylanase gene 1 in *Trichoderma reesei*

**DOI:** 10.1186/s13068-023-02417-w

**Published:** 2023-10-27

**Authors:** Wenqiang Xu, Yajing Ren, Yuxiao Xia, Lin Liu, Xiangfeng Meng, Guanjun Chen, Weixin Zhang, Weifeng Liu

**Affiliations:** 1 State Key Laboratory of Microbial Technology, Shandong University, No.72 Binhai Road, Qingdao, 266237 People’s Republic of China; 2 Shandong Lishan Biotechnology Co., LTD, Jinan, China

**Keywords:** Xylanase, Transcriptional repressor, *xyn1*, XYR1, Filamentous fungus

## Abstract

**Background:**

The well-known industrial fungus *Trichoderma reesei* has an excellent capability of secreting a large amount of cellulases and xylanases. The induced expression of cellulase and xylanase genes is tightly controlled at the transcriptional level. However, compared to the intensive studies on the intricate regulatory mechanism of cellulase genes, efforts to understand how xylanase genes are regulated are relatively limited, which impedes the further improvement of xylanase production by *T. reesei* via rational strain engineering.

**Results:**

To identify transcription factors involved in regulating xylanase gene expression in *T. reesei*, yeast one-hybrid screen was performed based on the promoters of two major extracellular xylanase genes *xyn1* and *xyn2*. A putative transcription factor named XTR1 showing significant binding capability to the *xyn1* promoter but not that of *xyn2*, was successfully isolated. Deletion of *xtr1* significantly increased the transcriptional level of *xyn1*, but only exerted a minor promoting effect on that of *xyn2*. The xylanase activity was increased by ~ 50% with XTR1 elimination but the cellulase activity was hardly affected. Subcellular localization analysis of XTR1 fused to a green fluorescence protein demonstrated that XTR1 is a nuclear protein. Further analyses revealed the precise binding site of XTR1 and nucleotides critical for the binding within the *xyn1* promoter. Moreover, competitive EMSAs indicated that XTR1 competes with the essential transactivator XYR1 for binding to the *xyn1* promoter.

**Conclusions:**

XTR1 represents a new transcriptional repressor specific for controlling xylanase gene expression. Isolation and functional characterization of this new factor not only contribute to further understanding the stringent regulatory network of xylanase genes, but also provide important clues for boosting xylanase biosynthesis in *T. reesei*.

**Supplementary Information:**

The online version contains supplementary material available at 10.1186/s13068-023-02417-w.

## Introduction

Lignocellulosic biomass is the most abundant renewable energy source on the earth that can be converted into fermentable sugars for production of biofuels and valuable chemicals. The major components of lignocellulosic biomass are cellulose (40–60%), hemicellulose (20–40%) and lignin (10–24%) [1]. Xylan accounts for a main constituent of hemicellulose and is frequently found to wrap around the outer layer of cellulose in lignocellulose. Its degradation is thus believed to greatly increase the accessibility of cellulose to cellulases, therefore, promoting the overall efficiency of lignocellulose hydrolysis [2]. Similar to cellulose, degradation of xylan requires a concerted action of a number of xylanolytic enzymes. Among others, endo-1,4-β-D-xylanase (EC3.2.1.8) (usually called xylanases) acts on the main chain backbone of xylan to release xylooligosaccharide and xylose that have found many industrial applications [3].

Filamentous fungi are the major contributors to lignocellulose degradation by producing a large amount of extracellular hydrolases. Among others, *Trichoderma reesei* is a well-known workhorse for production of cellulases and hemicellulases (mainly xylanases) and has a long history of safe use in industry [4–6]. The highest yield of *T. reesei* extracellular (hemi)cellulolytic enzyme cocktail is reported to reach up to 100 g/L [7]. However, the amount of extracellular xylanase in *T. reesei* is much lower than that of cellulase, implying a space to further increase the enzyme cocktail capability for lignocellulose hydrolysis and thus reduce enzyme cost by boosting xylanase production. Whereas *T. reesei* genome encodes five endo-β-1,4-xylanases XYNI-XYNV [8], more than 90% of extracellular xylanolytic activity is contributed by XYNI and XYNII, which thus represent the major xylanases of *T. reesei* [9]. Therefore, understanding the regulatory mechanism underlying the expression of these two major xylanases genes, *xyn1* and *xyn2* (xylanase gene 1 and 2), would greatly facilitate rational genetic engineering to improve *T. reesei* xylanase production.

Induced expression of genes encoding cellulases or xylanases in *T. reesei* is tightly controlled at the transcriptional level. While efforts made for dissecting the regulatory mechanism of xylanase genes largely fall behind those on cellulase genes in *T. reesei*, quite a few transcription factors have been found to be involved in controlling xylanase gene expression. The first class of transcription factors includes those co-regulating cellulase and xylanase genes, *e*.*g*., XYR1 [10–12], ACE2 [13, 14], and ACE3 [15, 16] acting as positive regulators, and CRE1 [17, 18] and ACE1 [18, 19] as negative regulators. Of these transcription factors, XYR1 acts as a crucial activator for both cellulase and xylanase genes since deletion of *xyr1* not only abrogates cellulase gene expression but also silences *xyn1* and *xyn2* [11, 12]. Consistently, it has been found that the putative XYR1-binding sites (5′-GGC(A/T)_3_–3′) identified from cellulase gene promoters are also distributed along the promoters of *xyn1* and *xyn2* [11], revealing a direct regulatory effect of XYR1 on the xylanase genes. The second class of transcription factors refers to those that specifically regulate xylanase genes but have hardly any effect on cellulase gene expression, consisting of only two repressors Xpp1 and SxlR. While Xpp1 obtained in a pull-down assay based on the *xyn2* promoter acts as a repressor for both *xyn1* and *xyn2* [20], SxlR plays an important role in inhibiting the transcription of *xyn1*, *xyn2*, and *xyn5* that code for xylanases belonging to glycoside hydrolase 11 family [21]. Despite this progress, a holistic picture of xylanase gene regulation entails the isolation and mechanistic characterization of more specific transcriptional regulators, which would definitely contribute to further understanding the molecular paradigm controlling xylanase expression in *T. reesei*.

In this study, a new transcription factor XTR1 was identified to have significant binding ability to the promoter of *xyn1* via yeast one-hybrid screen. Deletion of *xtr1* (jgi|Trire2|60132) markedly enhanced xylanase production with a predominant effect on the *xyn1* expression. The precise binding site of XTR1 within the *xyn1* promoter was identified and the core nucleotide region critical for XTR1 binding was determined. Further analyses indicated that XTR1 competes with the essential transactivator XYR1 for binding to the *xyn1* promoter.

## Results

### Identification of the *xtr1* gene encoding an *xyn1* promoter-binding protein

To identify putative transcriptional regulators of *xyn1* and *xyn2* that encode the two major extracellular xylanases in *T. reesei*, yeast one-hybrid screens were performed based on a 1.5 kb promoter of *xyn1* or *xyn2*, respectively. Each promoter excluding the putative core promoter region was further divided into two fragments to result in P*xyn1a* (− 750 ~ − 91 bp), P*xyn1b* (− 1500 ~ − 750 bp), P*xyn2a* (− 750 ~ − 43 bp) and P*xyn2b* (− 1500 ~ − 750 bp), respectively. P*xyn2a* was subsequently replaced with P*xyn2* (− 1500 ~ − 43 bp) due to the presence of self-activation activity (Additional file 1: Fig. S1). Each of the four promoter fragments was inserted into the pAbAi plasmid carrying the Aureobasidin A (AbA) resistance gene *AUR1-C*, which could only be activated when a prey fused to the GAL4 activation domain binds to the promoter fragment. The pAbAi plasmids carrying these promoter fragments were, respectively, transformed into yeast cells to generate bait strains. A *T. reesei* cDNA expression library prepared from xylanase-inducing conditions (with xylan as the carbon source) was transformed into each bait strain and the resulting transformants were selected on plates containing 100 ng/mL AbA. Several positive clones out of ~ 300 transformants were obtained and sequencing the prey plasmids revealed partial sequences encoding four independent *T. reesei* proteins. While Tr119805 binding to both P*xyn1a* and P*xyn2b* as well as Tr77279 binding to P*xyn1b* were predicted to be a hypothetical protein and a putative nuclear kinase, respectively, Tr103230 binding to P*xyn2* and Tr60132 binding to P*xyn1a* were both annotated as DNA-binding transcription factors.

**Fig. 1:**
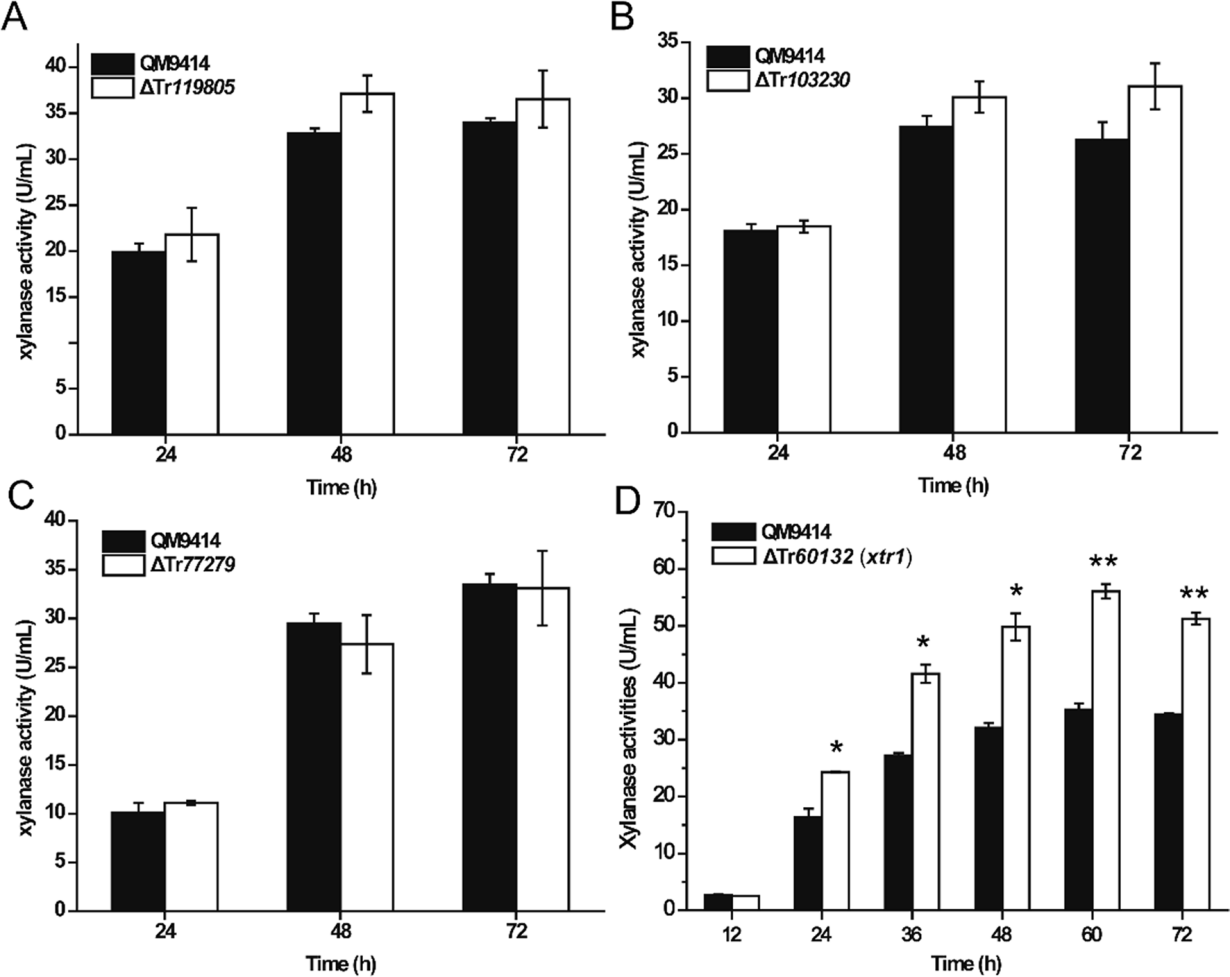
Deletion of *xtr1* markedly improved xylanase production. **A**–**D** Analyses of extracellular xylanase activities of the culture supernatant from the QM9414 and mutant strains including ΔTr119805 (**A**), ΔTr103230 (**B**), ΔTr77279 (**C**) and Δ*xtr1* (Tr60132) (**D**) cultivated on 0.5% (*w*/*v*) xylan for the indicated time periods. Values are the mean of three biological replicates. Error bars are the SD from these replicates. Significant differences (*t*-test *P < 0.05, **P < 0.01) were observed in extracellular xylanase activity between QM9414 and Δ*xtr1*

To test whether Tr119805, Tr103230, Tr60132 or Tr77279 play regulatory roles in xylanase production, their encoding genes were individually deleted in *T. reesei*. Determination of extracellular total xylanase activities demonstrated that, the absence of Tr119805, Tr103230, or Tr77279 had hardly any effect on xylanase production on xylan compared to the control strain QM9414 (Fig. 1A–C). By contrast, while elimination of Tr60132 exerted a neglectable effect on mycelial growth (Additional file 1: Fig. S2), it resulted in a ~ 50% increase in total xylan hydrolytic activities (Fig. 1D). Further analyses showed that Tr*60132* deletion hardly affected cellulase production, as determined by extracellular cellulolytic activities or the relative transcriptional levels of the main cellulase genes (Additional file 1: Fig. S3), indicating that Tr60132 exerts specific regulatory effect on xylanase production. Tr60132 was hereafter named XTR1 for xylanase gene transcriptional repressor 1.

**Fig. 2:**
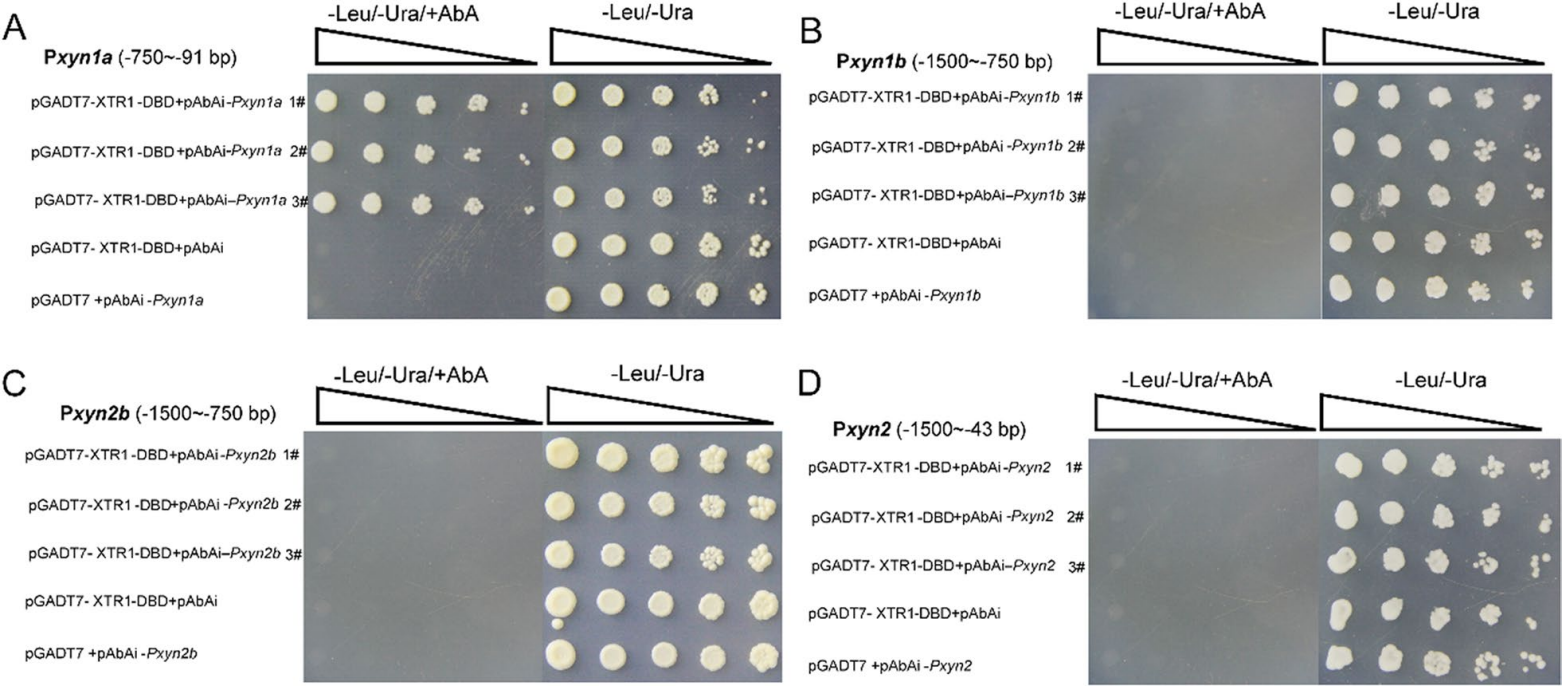
XTR1 DBD binds to the *xyn1* promoter. **A**–**D** Yeast one-hybrid analyses of binding ability of XTR1 DBD to the *xyn1* or *xyn2* promoter fragments including P*xyn1a* (− 750 ~ − 91 bp) (**A**), P*xyn1b* (− 1500 ~ − 750 bp) (**B**), P*xyn2b* (− 1500 ~ − 750 bp) (**C**), and P*xyn2* (− 1500 ~ − 43 bp) (**D**). Serial dilutions of yeast transformant cells harboring the indicated plasmids were spotted on double dropout medium (-Leu/-Uracil) with or without AbA supplement, and were allowed to grow at 28 °C for 3 days. Yeast cells carrying pGADT7 with XTR1 DBD encoding sequence plus empty pAbAi or those with empty pGADT7 plus pAbAi harboring the indicated promoter fragments were set as negative controls

Sequence analyses revealed that XTR1 annotated as a fungal transcription factor, contains a typical Cys_2_-His_2_ zinc finger-type DNA binding domain (DBD) at its C terminus (285 ~ 535 aa). Yeast one-hybrid assay verified the significant binding ability of XTR1 DBD to the promoter fragment P*xyn1a* (− 750 ~ − 91 bp) (Fig. 2B), but not to P*xyn1b* (− 1500 ~ − 750 bp) or P*xyn2* fragments (− 1500 ~ − 750 bp or 1500 ~ − 43 bp) (Fig. 2C–E). These results were consistent with those from yeast one-hybrid screening analysis.

**Fig. 3:**
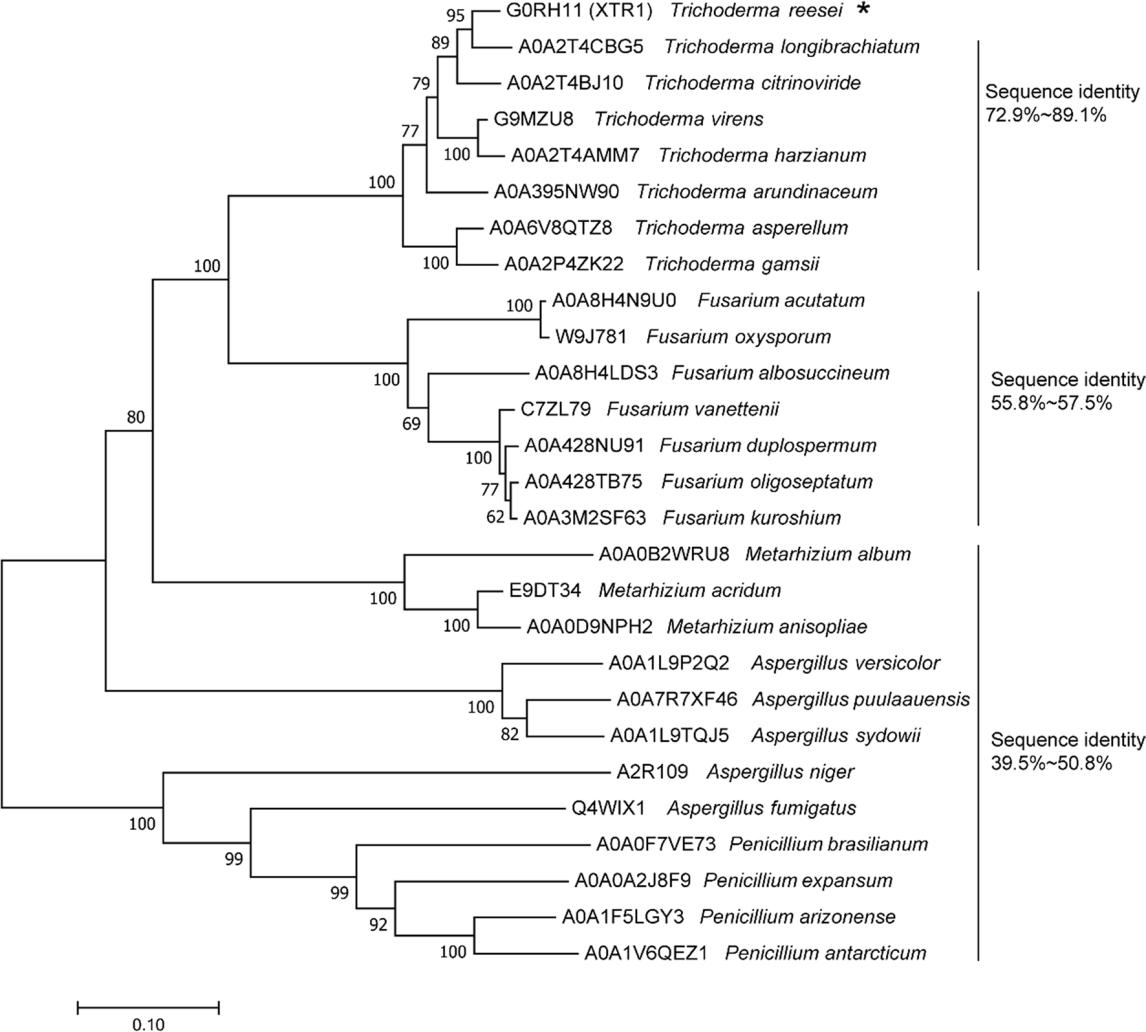
Phylogenetic analysis of XTR1 and its fungal orthologues. XTR1 from *T. reesei* was indicated with an asterisk. The entry numbers of these sequences retrieved from the Uniprot database were indicated. The sequence identities between XTR1 and its orthologues were also shown. Amino acid sequence alignment was performed using ClustalW [35]. Phylogenetic analysis was performed with MEGA7.0 [36] using the Neighbor-joining method with 1000 bootstraps

Phylogenetic analyses further revealed that orthologues of XTR1 are wildly distributed in a plethora of filamentous fungi (Fig. 3), including *Trichoderma*, *Fusarium*, *Metarhizium*, *Aspergillus* and *Penicillium* species. However, none of these orthologues has been assessed regarding their function in the production of hemicellulolytic or cellulolytic hydrolases.

### XTR1 acts as a nuclear transcriptional repressor of *xyn1*

**Fig. 4:**
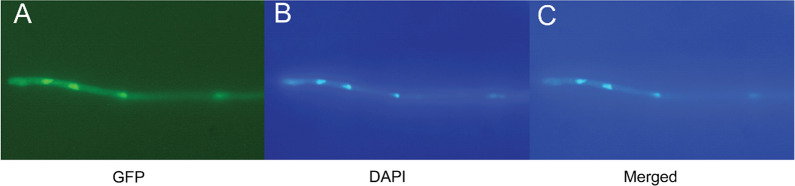
Analysis of subcellular localization of XTR1. **A** Fluorescence microscopic analyses of CpΔ*xtr1* expressing XTR1-GFP (Green) after cultivation on 1% glucose for 16 h. **B** Fluorescence microscopic analyses of CpΔ*xtr1* cells stained with DAPI (Blue). **C** Merged image of (**A**) and (**B**)

To further ascertain the repressive role of XTR1 in xylanase production and determine the subcellular localization of XTR1, Δ*xtr1* was complemented with an expression cassette of *xtr1* fused to *gfp* (green fluorescence protein encoding gene) under the control of a constitutively strong promoter *tcu1* [22] which resulted in an overexpression of *xtr1* as compared to that of QM9414 strain (yielding Cp∆*xtr1*) (Additional file 1: Fig. S4). Fluorescence signal in Cp∆*xtr1* was found to be mainly accumulated in the nucleus, implicating that XTR1 is a nuclear protein (Fig. 4).

**Fig. 5:**
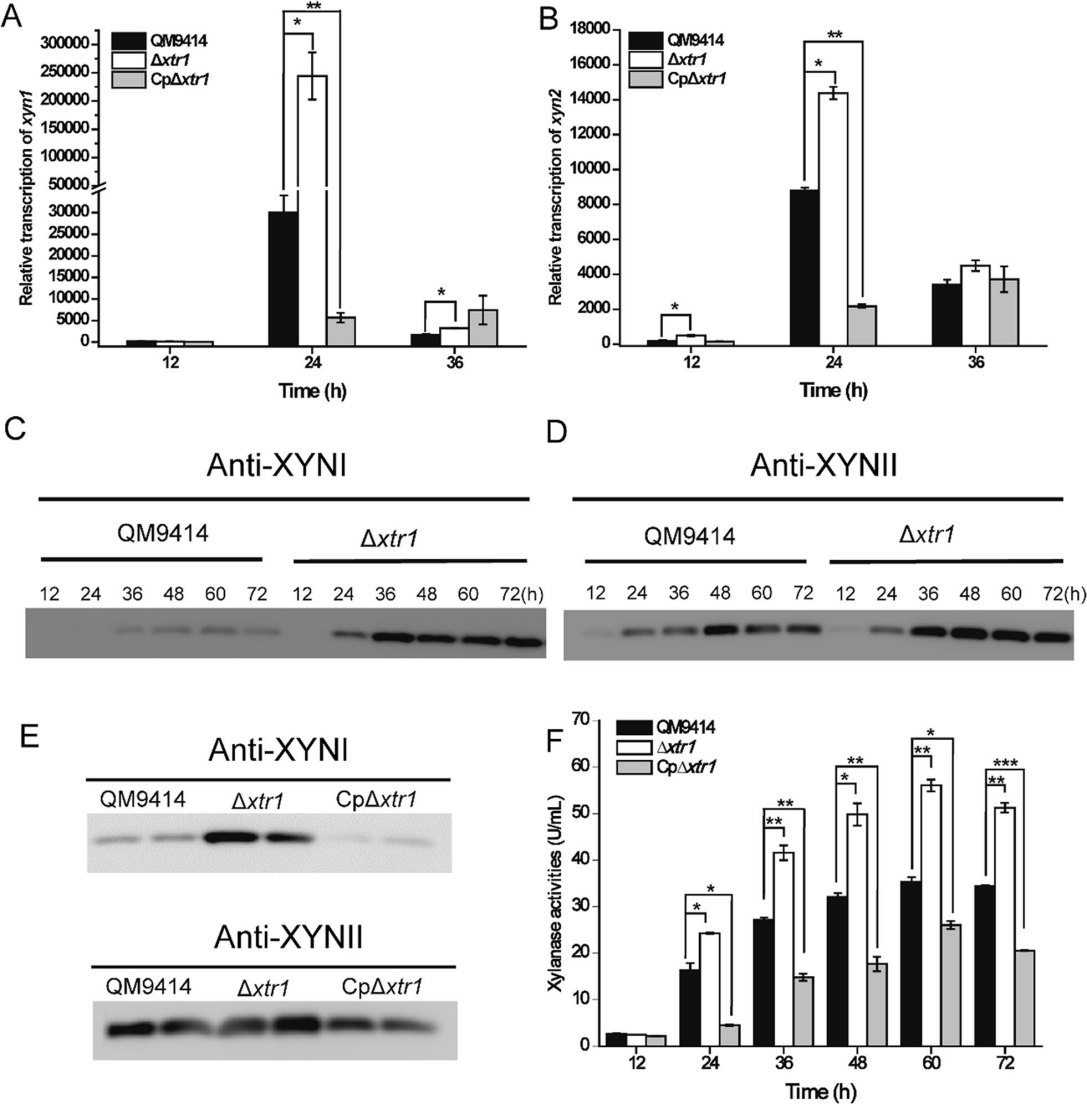
XTR1 exerts a predominant regulatory effect on the transcription of *xyn1*. **A**, **B** Quantitative RT-PCR analyses of the relative transcription of *xyn1* (**A**) and *xyn2* (**B**) in QM9414, Δ*xtr1* and CpΔ*xtr1* cultivated on 0.5% xylan. **C**, **D** Western blot analyses of XYNI (**C**) and XYNII (**D**) production in the culture supernatant of QM9414 and Δ*xtr1* that were cultivated on xylan for the indicated time periods. **E** Western blot analyses of XYNI (upper) and XYNII (lower) production in the culture supernatant of QM9414, Δ*xtr1* and CpΔ*xtr1* cultivated on xylan for 60 h. Two sample repeats for each strain were shown. **F** Extracellular xylanase activity analyses of the culture supernatant from QM9414, Δ*xtr1* and CpΔ*xtr1* cultivated on xylan. Values are the mean of three biological replicates. Error bars are the SD from these replicates. Significant differences (*t*-test *P < 0.05, **P < 0.01, *** P < 0.001) were observed in the relative transcription level of *xyn1* and *xyn2* and extracellular xylanase activities between QM9414 and Δ*xtr1* or CpΔ*xtr1*. Equal volume of culture supernatant (15 μL) of the relevant strains was applied for western blot analyses as shown in (**C**–**E**)

Given the potential of XTR1 as a DNA-binding factor to regulate expression of *xyn1*, the effect of *xtr1* deletion and overexpression on the transcriptional expression of *xyn1* was performed using quantitative real-time PCR (qRT-PCR) analyses. Results showed that deletion of *xtr1* causedup to eightfold-increase in the relative transcriptional level of *xyn1* after an induction period of 24 h on xylan (Fig. 5A). Of note, the remarkable elevation in the relative transcription of *xyn1* was fully reverted in Cp∆*xtr1* to a level much lower than that of QM9414 (Fig. 5A), indicating that XTR1 overexpression exerted a marked inhibitory effect on *xyn1* transcription. The transcriptional level of *xyn2* was slightly increased (0.64-fold) in Δ*xtr1* but remarkably downregulated in Cp∆*xtr1* as compared with that of QM9414 (Fig. 5B). Moreover, Western blot analyses were performed to detect the extracellular protein levels of XYNI and XYNII using their respective antibodies. In agreement with transcriptional analyses, XYNI production by ∆*xtr1* not only occurred earlier but was also significantly elevated, while only a minor increase was observed in the production of XYNII (Fig. 5C, D). Western blot assays also revealed a remarkable reduction in extracellular production of XYNI and a moderate decrease in XYNII production in Cp∆*xtr1*, as compared with ∆*xtr1* or QM9414 (Fig. 5E). As expected, the extracellular total xylanase activity in Cp∆*xtr1* was markedly decreased compared to that of QM9414 or ∆*xtr1* (Fig. 5F), whereas Cp∆*xtr1* exhibited similar mycelial growth to that of QM9414 or ∆*xtr1* on xylan (Additional file 1: Fig. S2C). Altogether the results indicate that XTR1 acts as a transcriptional repressor for xylanase genes in *T. reesei*, most probably with a direct and predominant role in regulating the expression of *xyn1*.

### Identification of the XTR1 binding site in the *xyn1* promoter

**Fig. 6:**
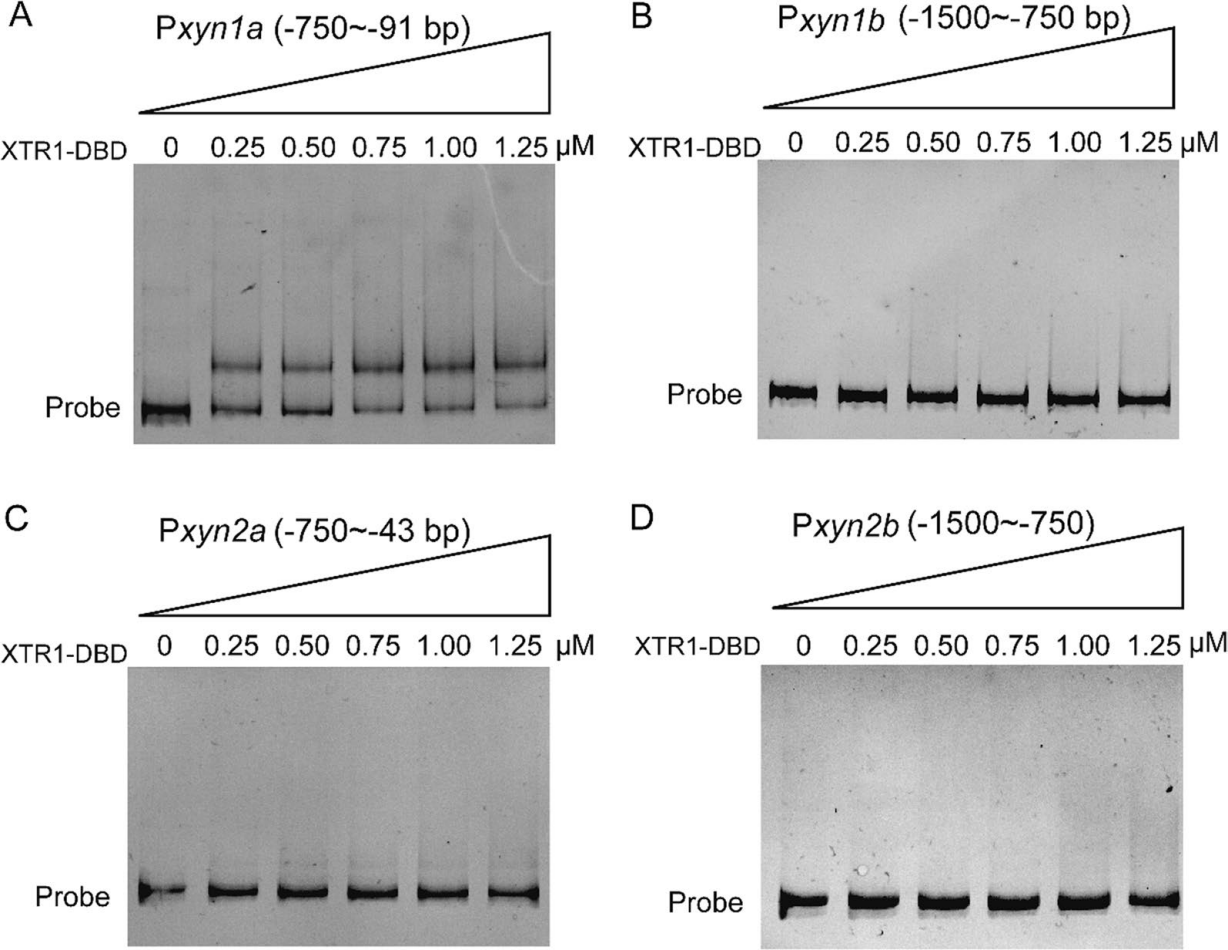
EMSAs of XTR1 binding to the promoter fragments of *xyn1* and *xyn2*. **A**–**D** The promoter fragments of P*xyn1a* (− 750 ~ − 91 bp) (**A**), P*xyn1b* (− 1500 ~ − 750 bp) (**B**), P*xyn2a* (− 750 ~ − 43 bp) (**C**), and P*xyn2b* (− 1500 ~ − 750 bp) (**D**) were, respectively, incubated with the purified recombinant XTR1 DBD protein and subjected to electrophoresis analyses

**Fig. 7:**
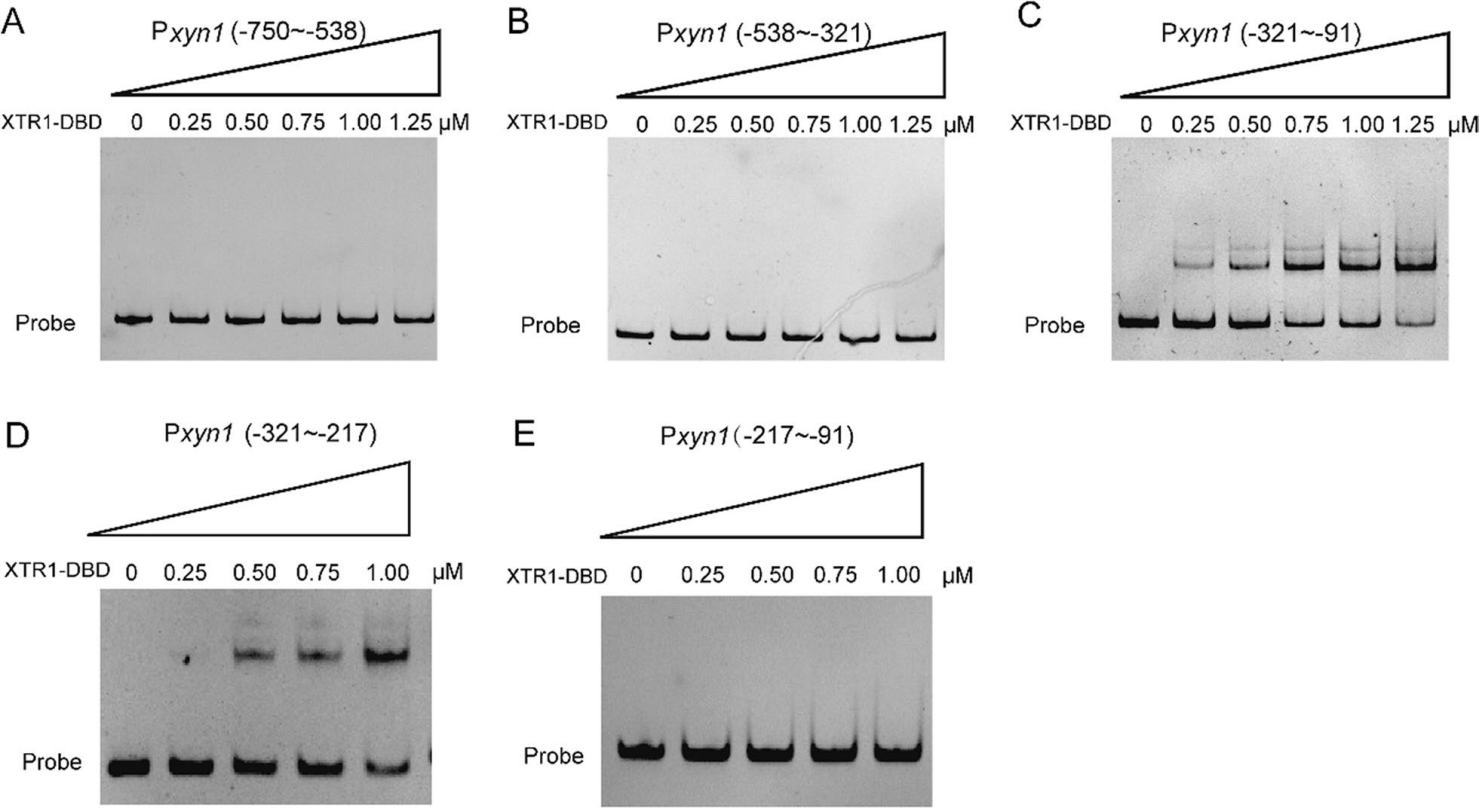
EMSAs of XTR1 binding to the promoter fragments of *xyn1*. **A**–**E** The promoter fragments of P*xyn1* (− 750 ~ − 538 bp) (**A**), P*xyn1* (− 538 ~ − 321 bp) (**B**), and P*xyn1* (− 321 ~ − 91 bp) (**C**), P*xyn1* (− 321 ~ − 217 bp) (**D**), and P*xyn1* (− 217 ~ − 91 bp) (**E**) were, respectively, incubated with the purified recombinant XTR1 DBD protein and subjected to electrophoresis analyses

**Fig. 8:**
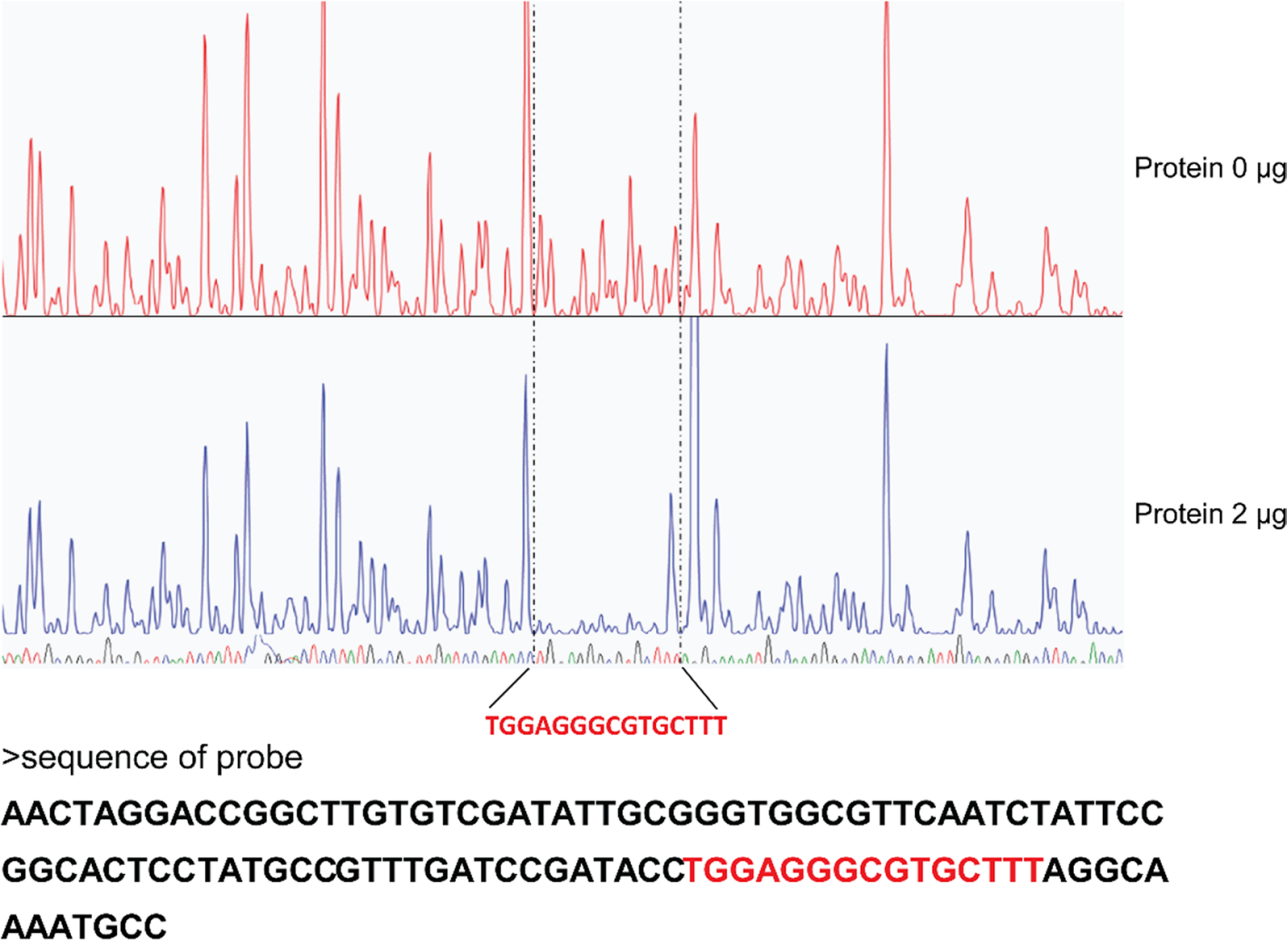
Determination of the precise binding motif of XTR1 via DNase I footprinting analysis. The promoter fragment P*xyn1* (− 321 ~ − 217 bp) was used as the probe and its sequence was shown, wherein the nucleotide sequence protected by recombinant XTR1-DBD was indicated in red

To investigate the direct binding of XTR1 to the *xyn1* promoter, the C-terminal DNA binding domain (DBD) of XTR1 (XTR1-DBD) was heterologously expressed in *E. coli* and purified for in vitro electrophoretic mobility shift assays (EMSAs). While no obvious motility shift was observed with P*xyn1b* or the *xyn2* promoter fragments, significant binding was detected to P*xyn1a* (Fig. 6). These results exactly recapitulated the observations obtained from yeast one-hybrid assays (Fig. 2), supporting that XTR1 functions as a direct transcriptional regulator for *xyn1*. To precisely pinpoint the XTR1 binding site in the *xyn1* promoter, P*xyn1a* was first divided into three segments including P*xyn1* (− 750 ~ − 538 bp), P*xyn1* (− 538 ~ − 321 bp), and P*xyn1* (− 321 ~ − 91 bp) and EMSAs with these segments showed that XTR1-DNA complex was only formed with P*xyn1* (− 321 ~ − 91 bp) (Fig. 7A–C). This region was further divided into two shorter ones, P*xyn1* (− 321 ~ − 217 bp) and P*xyn1* (− 217 ~ − 91 bp), and XTR1-DBD was found to bind P*xyn1* (− 321 ~ − 217 bp) but not P*xyn1* (− 217 ~ − 91 bp) (Fig. 7D-E). In a DNase I footprinting assay using P*xyn1* (− 321 ~ − 217 bp), an apparently protected sequence (TGGAGGGCGTGCTTT) was identified when up to 2 μg of XTR1-DBD was applied (Fig. 8).

**Fig. 9:**
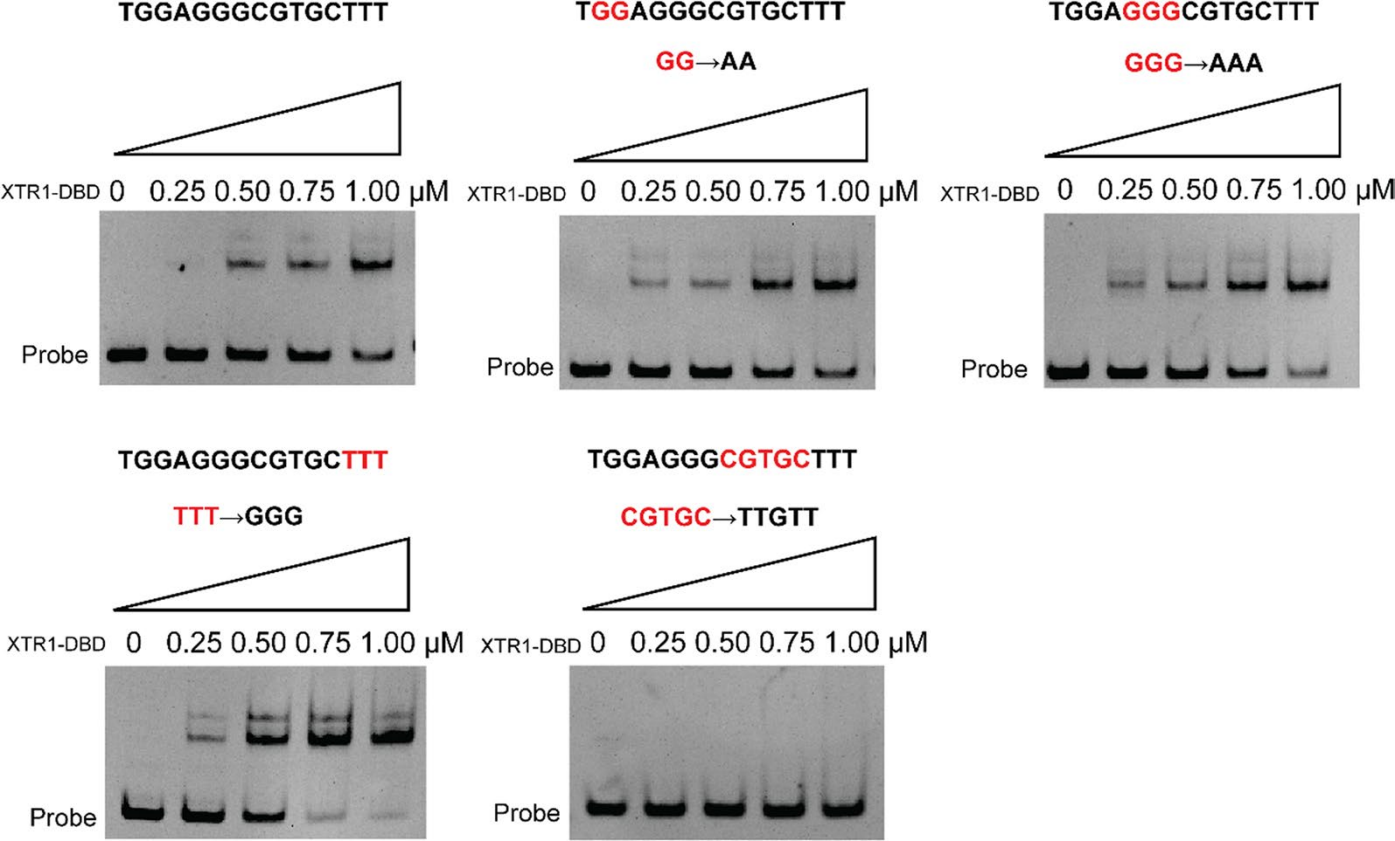
Determination of the critical nucleotides within *xyn1* promoter for XTR1 binding via EMSAs. The P*xyn1* (− 321 ~ − 217 bp) fragments with the original or changed nucleotide sequence in the binding sites of XTR1 as shown were, respectively, incubated with the purified recombinant XTR1 DBD protein and subjected to EMSAs. The mutated nucleotides were indicated in red

To define specific nucleotides crucial for XTR1-DBD binding, the effect of mutations of the protected sequence nucleotides was examined by EMSAs. When the GG at positions 2 and 3 were replaced with AA or the GGG at positions 5–7 were replaced with AAA, formation of complexes of XTR-DBD and DNA probes was still observed (Fig. 9A–C), indicating that the binding ability of XTR1-DBD to the promoter probe was not impaired by these changes. Interestingly, mutation of the 3’ end TTT into AAA somehow increased the binding activity of XTR1-DBD. In contrast, a complete loss of XTR1-DBD binding was observed when CGTGC at positions 8–12 were mutated to TTGTT (Fig. 9D, E), revealing that these nucleotides represent the core sequence for XTR1-DBD binding to the *xyn1* promoter.

### XTR1 competes with the transactivator XYR1 for binding to the *xyn1* promoter

**Fig. 10:**
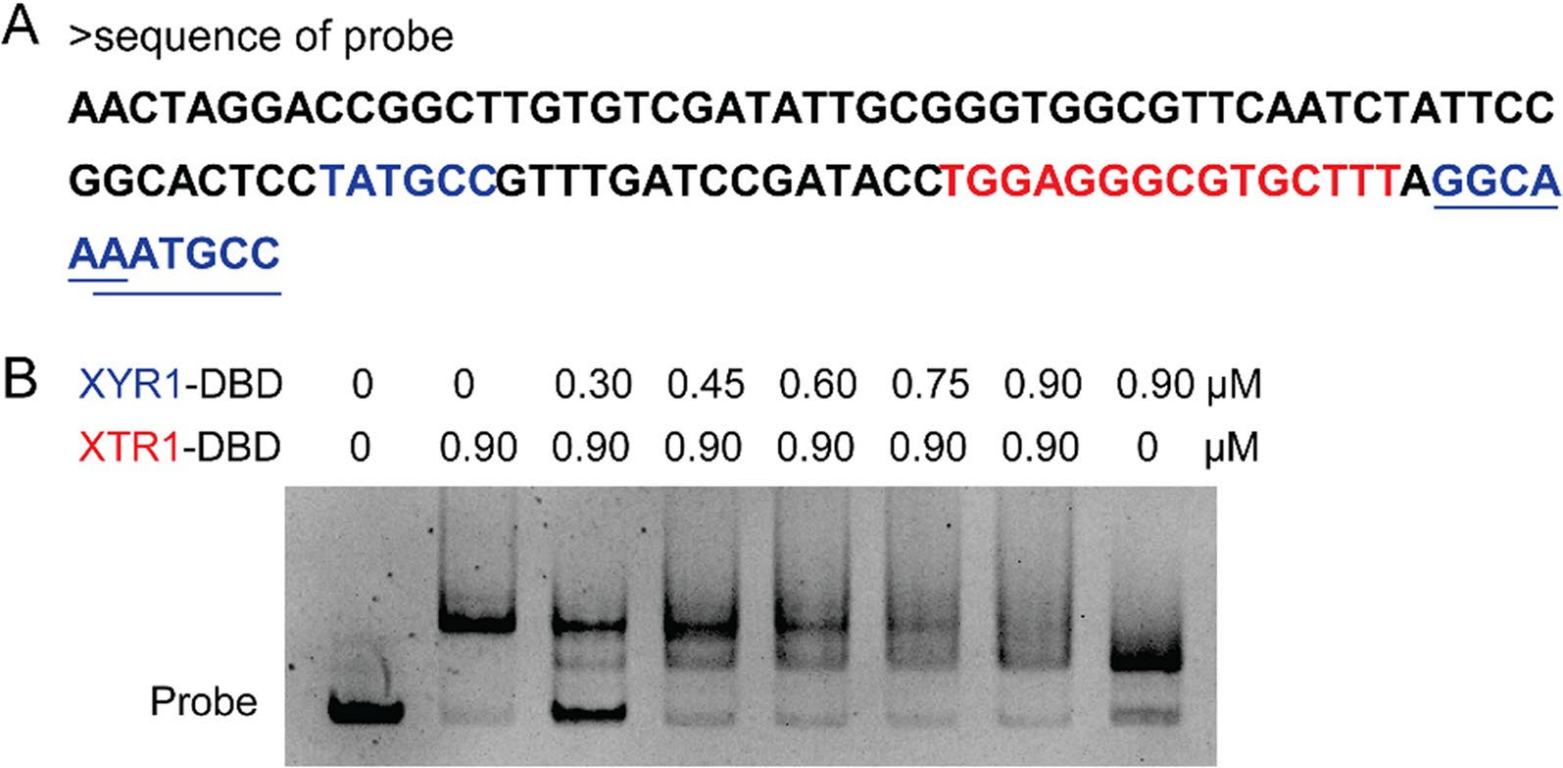
XTR1 competes with the transactivator XYR1 for binding to *xyn1* promoter. **A** The sequence of P*xyn1* (− 321 ~ − 217 bp) wherein three putative binding sites of the transactivator XYR1 was indicated in blue, and the binding site of the repressor XTR1 identified in this study was indicated in red. Two inverted putative binding sites of XYR1 were also underlined for clarity. **B** Competitive EMSA with the presence of both recombinant XYR1 DBD and XTR1 DBD. The P*xyn1* (− 321 ~ − 217 bp) fragment was used as the probe

Previous studies demonstrate that transcription of *xyn1* is positively regulated by XYR1, the essential transactivator for almost all the cellulase and hemicellulase genes in *T. reesei* [10, 18]. Given that XYR1 has the binding consensus sequence of 5′-GGC(A/T)_3_–3′ [11], three putative XYR1-binding sites including 5′-TATGCC-3′, 5′-GGCAAA-3′, and 5′-AATGCC-3′ were found to exist within the P*xyn1* (− 321 ~ − 217 bp) fragment, with two of them being adjacent to the identified binding site of XTR1 (Fig. 10A). Consistently, recombinant DBD protein of the transactivator XYR1 showed apparent binding capability to P*xyn1* (− 321 ~ − 217 bp), forming a band shift in EMSA distinct from that yielded by XTR1 binding (Fig. 10B). To figure out whether a competition might exist between XYR1 and XTR1 in binding to the promoter, a competitive EMSA assay employing different ratios of recombinant XYR1 and XTR1 DBDs was performed with P*xyn1* (− 321 ~ − 217 bp) as probe. When XTR1 DBD was held at a constant level (0.9 μM) and XYR1 DBD was increased from 0 to 0.9 μM, the XTR1-DNA complex gradually disappeared and the XYR1-DNA complex appeared (Fig. 10B). These results suggest that XTR1 might repress *xyn1* expression via preventing the transactivator XYR1 from binding to the *xyn1* promoter.

## Discussion

Induced expression of genes encoding cellulases and xylanases in *T. reesei* is tightly controlled at the transcriptional level involving a plethora of transcription factors. Identification and functional characterization of these transcription factors would greatly contribute to not only understanding the underlying regulatory network controlling their induced biosynthesis, but also improving the production of cellulases and xylanases through genetic engineering. However, study on the detailed aspects of transcriptional regulation of xylanase genes in *T. reesei* is lagging behind those of cellulase genes, which impedes the further improvement of xylanase yield and thus the efficacy of the whole extracellular enzyme cocktail for lignocellulose hydrolysis. In the present study, we identified a transcription factor Tr60132 (named XTR1 here) that exhibits significant binding ability to the *xyn1* promoter. Although orthologues of XTR1 are widely distributed in a number of fungal species, especially those capable of secreting cellulases and xylanases, none of them has been functionally characterized previously. The observation that elimination of XTR1 in *T. reesei* markedly increased extracellular xylanase activity by ~ 50%, but had hardly any effect on cellulase synthesis, indicated that XTR1 represents a new transcription factor specifically regulating xylanase gene expression*.*

Whereas XYNI and XYNII with quite similar enzyme characteristics are responsible for the majority of secreted xylanase activities in *T. reesei* [9], expression of their respective encoding genes *xyn1* and *xyn2* does not follow exactly the same regulatory pattern by involving distinct transcription factors [23]. In particular, the well-known carbon catabolite repressor CRE1 and the transcriptional repressor ACE1 directly binds to the promoter of *xyn1* but not that of *xyn2* [18, 24]. On the other hand, the activator ACE2 is not involved in the regulation of *xyn1* expression but contributes to the basal and induced expression of *xyn2* via binding to the xylanase activating element (XAE) in the *xyn2* promoter [13, 14, 18, 25]. In this study, both yeast one-hybrid and EMSA analyses demonstrated that XTR1 only binds the promoter of *xyn1* but not that of *xyn2*. In accordance with these binding results, either XTR1 elimination or overexpression exerted a more dominant effect on the *xyn1* expression. Moreover, the nucleotide sequence bound by XTR1 in the *xyn1* promoter as determined via DNase I footprinting assay was not found within the *xyn2* promoter. All these results implicated that XTR1 acts as a transcription factor specifically for *xyn1* regulation. The minor effect on XYNII production resulting from XTR1 elimination or overexpression was probably due to an indirect impact ensuing from changes in *xyn1* expression on either the transformation of xylanase-inducing signals or other as yet unknown factors involved in regulating xylanase gene expression, which still needs future investigation. Nonetheless, our study on XTR1 provide insights into further understanding the regulatory mechanism of *xyn1* as well as the differential regulatory strategies used for modulating *xyn1* and *xyn2* expression in *T. reesei*.

Similar to Xpp1 and SxlR isolated previously [20, 21], XTR1 acts as a repressor to inhibit the transcription of *xyn1*. Besides these transcriptional repressors, two transactivators for cellulase genes, XYR1 and ACE3, are required for efficient induction of xylanase genes [10, 15]. Unlike ACE3 whose binding site is not present in the *xyn1* or *xyn2* promoter [16], XYR1-binding sites are substantially distributed within the two promoters [11], demonstrating a direct regulatory role of XYR1 in *xyn1* and *xyn2* expression. As for *xyn1*, a total of sixteen putative XYR1-binding sites are present within its promoter [11], and three of them were located in the proximity to the identified binding site of XTR1. Possibility, therefore, exists that XTR1 might exert its inhibitory effect just by preventing XYR1 from binding to the same promoter region. Our competitive EMSAs indeed support that XTR1 competes with XYR1 for binding to the promoter fragment of P*xyn1* (− 321 ~ − 217 bp). This competition, however, would not completely block the induced *xyn1* expression under physiological conditions largely due to the binding of XYR1 to other sites in the promoter. When XTR1 was absent, more occupancy by XYR1 at the indicated promoter sites would probably synergize with other transcription factors including XYR1 itself bound at other promoter regions to elevate the transcriptional expression of *xyn1*. It should be noted that mechanistic details other than competitive binding regarding the repressive action of XTR1 cannot be excluded. Notwithstanding this, our results revealing the competitive binding between XTR1 and XYR1 as well as determining the core nucleotide sequence of XTR1 binding motif in the *xyn1* promoter would provide important clues for rational strain engineering of *T. reesei* to further increase xylanase production. The anticipated engineering strategies include but are not limited to elimination of the XTR1 binding site and simultaneously increasing the binding sites for XYR1 in the xylanase gene promoter as well as overexpression of XYR1 in the *xtr1* deletion strain.

## Conclusions

XTR1 was identified as a new transcriptional repressor specifically controlling xylanase gene 1 expression in *T. reesei*. Elimination of XTR1 improved total xylanase activity by 50%. Results in this study not only provide insights into the stringent regulatory network for xylanase gene expression, but also demonstrate that XTR1 represents a prospective target candidate for genetic engineering to enhance xylanase production in *T. reesei*.

## Methods

### Strains, media, and culture conditions

*T. reesei* QM9414 (ATCC 26921), a cellulase-enhanced derivative of the original strain QM6a, was used as the control strain throughout the study. QM9414-Δ*pyr4* with the uridine trophic marker gene *pyr4* deleted [26] was used as the parental strain. *T. reesei* strains were maintained on malt extract agar. For analyses of gene transcription and enzyme production, *T. reesei* were pre-cultured at 28 °C for 24 h in 500 mL-flask containing 150 mL Mandels-Andreotti (MA) medium (17.907 g Na_2_HPO_4_·12H_2_O, 2 g K_2_HPO_4_, 1.4 g (NH_4_)_2_SO_4_, 0.3 g urea, 0.15 g MgSO_4_·7H_2_O, 0.15 g CaCl_2_, 0.005 g FeSO_4_·7H_2_O, 0.0016 g MnSO_4_·H_2_O, 0.0014 g ZnSO_4_·7H_2_O, 0.002 g CoCl_2_·2H_2_O and 0.5 mL Tween-80 per liter, pH 5.0) supplemented with 1% (*v*/*v*) glycerol as carbon source. The mycelia were then harvested via filtration and washed twice with MA medium without any carbon source. Equal amounts of mycelia were transferred to 150 mL of fresh MA medium containing 0.5% (*w*/*v*) beechwood xylan (Biosynth Carbosynth, United Kingdom) or 1% (*w*/*v*) Avicel cellulose (Sigma-Aldrich, United States) as the carbon source, and were cultivated for the indicated time periods.

*Escherichia coli* DH5a was used for routine plasmid construction and *E. coli* Origami BL21 (DE3) cells were used as a host for the heterologous production of XYR1 DBD or XTR1 DBD proteins. Both strains were cultured in lysogeny broth in a rotary shaker (200 rpm) at 37 ℃.

The *Saccharomyces cerevisiae* strain Y1Hgold (*MATα, ura3-52, his3-200, ade2-101, trp1-901, leu2-3, 112, gal4Δ, gal80Δ, met-,* MEL1) was used as the host for the one-hybrid screen. Yeast cells were routinely cultivated in YPD medium (1% yeast extract, 2% peptone and 2% glucose) at 28 ℃. Synthetic Complete (SC) medium lacking uracil and leucine with 100 ng/mL of AbA was used for positive clone selection.

### Yeast one-hybrid analyses

To identify *T. reesei* proteins binding to *xyn1* or *xyn2* promoter via yeast one-hybrid screen, the 1.5 kb-*xyn1* promoter upstream the initiation codon ATG of *xyn1* with the putative core promoter region removed was divided into two parts: P*xyn1a* (− 750 ~ − 91 bp) and P*xyn1b* (− 1500 ~ − 750 bp). Similarly, the 1.5 kb-*xyn2* promoter was divided into P*xyn2a* (− 750 ~ − 43 bp) and P*xyn2b* (− 1500 ~ − 750 bp). All the above four fragments as well as P*xyn2* (− 1500 ~ − 43 bp) were amplified from QM9414 genomic DNA, and were, respectively, inserted into the pAbAi plasmid (Clontech, United States) to obtain the bait plasmids including pAbAi-P*xyn1a* (− 750 ~ − 91 bp), pAbAi-P*xyn1b* (− 1500 ~ − 750 bp), pAbAi-P*xyn2a* (− 750 ~ − 43 bp), pAbAi-P*xyn2b* (-1500 ~ -750 bp), and pAbAi-P*xyn2* (− 1500 ~ − 43 bp). These bait plasmids were, respectively, transformed into *S*. *cerevisiae* Y1Hgold to obtain bait strains. Since the bait strain harboring pAbAi-P*xyn2a* (− 750 ~ − 43 bp) could readily grow in the presence of 100 ng/mL AbA, it was not applied for subsequent test. Therefore, four bait strains harboring pAbAi-P*xyn1a* (− 750 ~ − 91 bp), pAbAi-P*xyn1b* (− 1500 ~ − 750 bp), pAbAi-P*xyn2b* (− 1500 ~ − 750 bp), or pAbAi-P*xyn2* (− 1500 ~ − 43 bp) were applied for yeast one-hybrid screen.

For preparation of the cDNA library, mycelia of QM9414 cultivated on 0.5% (w/v) xylan for 9 h were harvested and subjected to RNA extraction using the TRIzol reagent (Invitrogen, United States). A cDNA library was constructed by ligating the reverse transcribed cDNA fragments (synthesized using SMART cDNA Library Construction Kit (TaKaRa, Janpan)) into the pGADT7 plasmid according to the Matchmaker one-hybrid system manual (Clontech, United States). Ten microgram of the constructed *T. reesei* cDNA library plasmids were then transformed into the above four bait strains, respectively. Yeast transformants were selected on SC plates lacking uracil and leucine but containing 100 ng/mL AbA. The colonies were picked and the harbored plasmids were sequenced.

To test the binding ability of XTR1 DBD to *xyn1* promoter fragments, the nucleotide sequence encoding XTR1 DBD (amino acids 285 ~ 535) was amplified from the genomic DNA of QM9414 and inserted into pGADT7, which was subsequently transformed into the four bait strains as described above. The resulting transformants were collected, serially diluted, and spotted on double dropout medium (-Leu/-Uracil) with or without AbA supplement and were allowed to grow at 28 °C for 3 days. Yeast cells expressing pGADT7 carrying XTR1 DBD plus empty pAbAi or those with empty pGADT7 plus pAbAi harboring the indicated promoter fragments were used as negative controls.

### Construction of* T. reesei* mutant strains

To delete Tr60132 (*xtr1*), Tr119805, Tr103230, or Tr77279, the upstream and downstream noncoding regions (~ 2.0 kb) of each gene were amplified from QM9414 genomic DNA, and ligated into pDonor*pyr4* [27] via BP-cloning (Invitrogen, United States) to yield the deletion plasmids, which were, respectively, transformed into *T. reesei* QM9414-Δ*pyr4* [26] to generate the corresponding deletion strains.

To complement Δ*xtr1* and detect the subcellular localization of XTR1, the full-length *xtr1* sequence without stop codon was amplified from QM9414 genomic DNA and fused to *gfp* via overlap-extension PCR [28]. The fused fragment was inserted into the pMDP*tcu1*-T*trpC*-*hph* [29] to yield the complementation plasmid. This plasmid was transformed into Δ*xtr1* to result in Cp∆*xtr1*, wherein *xtr1*-*gfp* was expressed under the control of the constitutively strong *tcu1* promoter [22, 30]. All the primers used were listed in Additional file 1: Table S1.

Transformation of *T. reesei* was performed as previously described [31]. The transformants were selected on minimal medium for either uridine prototroph or for resistance to hygromycin (120 μg/mL). Anchored PCR was used to verify the correct integration events.

### Growth assays

To test mycelial growth on agar plates, *T. reesei* strains were pre-cultured on minimal media agar plate with 1% (*w*/*v*) glucose as carbon source for two days and then a slice of same-size circle area (about 1 cm^2^) covered with mycelia of the corresponding strain was taken and inoculated on minimal media agar plate supplemented with glucose, lactose, xylose, or xylan at a final concentration of 1% (*w*/*v*). Colony diameters were measured after plate incubation at 28 ℃ for three days. To analyze the growth in liquid medium, *T. reesei* strains were pre-cultured at 28 °C for 36 h in MA medium supplemented with 1% (*v*/*v*) glycerol. Mycelia were collected through filtration and washed twice with medium without any carbon source, then equal amounts of mycelia were transferred to fresh MA with 0.5% (*w*/*v*) beechwood xylan (Biosynth Carbosynth, United Kingdom) as the sole carbon source. After cultivation for the indicated periods, mycelia were filtered, dried at 80 ℃, and weighed for biomass quantification.

### Fluorescence microscopy analysis

To visualize XTR1-GFP, spores of Cp∆*xtr1* were inoculated and germinated in minimal medium containing 1% (*w*/*v*) glucose for 16 h-cultivation and the mycelia were subjected to fluorescence microscopy analyses. For nucleus visualization, DAPI (4’,6-diamidino-2-phenylindole dihydrochloride) staining was performed as previously reported [32]. The XTR1-GFP or DAPI fluorescence was detected with a Nikon Eclipse 80i fluorescence microscope (Nikon, Melville, NY, United States), and the images were captured and processed by NIS-ELEMENTSAR software.

### Enzyme activity and protein analyses

Xylanase activities were determined using beechwood xylan (Biosynth Carbosynth, United Kingdom) as substrate by measuring the amount of xylose released. Briefly, the assay was carried out in a 120 µL-reaction mixture containing 60 µL of diluted culture supernatant and 60 µL of 0.5% (*w*/*v*) xylan dissolved in 50 mM sodium acetate buffer (pH 4.8). The reaction mixture was incubated at 50 ℃ for 10 min and the release of reducing sugar was determined using DNS method with xylose as standard. One unit of xylanase activity was defined as the amount of enzyme capable of releasing 1 µmol of xylose per minute. Determination of cellulolytic activities including cellobiohydrolase, β-glucosidase and endoglucanase activities were performed as previously described [33].

SDS-PAGE and western blot were performed according to standard protocols. XYNI and XYNII were immunoblotted using polyclonal antibodies raised against peptides of XYNI (amino acids of 52 ~ 65) and XYNII (amino acids of 79 ~ 92) [34], respectively.

### Quantitative real-time PCR (qRT-PCR)

Total RNA was extracted using TRIzol reagent (Sangong Biotech, China) and purified using the TURBO DNA-free kit (Ambion, United States) to remove gDNA according to the manufacturer’s instructions. Reverse transcription was performed using the PrimeScript RT reagent Kit (Takara Bio, Japan) according to the instructions. Quantitative PCR was performed on a Bio-Rad myIQ2 thermocycler (Bio-Rad, United States). Data were analyzed using the relative quantitation/comparative CT (ΔΔCT) method and were normalized to an endogenous control (*actin*). Three biological replicates were performed for each analysis and the results. Statistical analysis was performed using the student’s *t*-test analysis.

### Recombinant protein production in *E. coli*

For heterologous expression of XTR1 DBD in *E. coli*, the DNA fragment encoding XTR1 DBD (amino acids 285 ~ 535) was amplified from QM9414 genomic DNA and was inserted into *Nde*I/*Xho*I-digested pET22b( +) to obtain the pET22b-XTR1_285-535_ plasmid. The pET22b-XYR1_1-195_ plasmid used for expression of XYR1 DBD (amino acids 1 ~ 195) was constructed as previously described [32].

To purify XYR1_1~195_ and XTR1_185-535_, *E. coli* Origami BL21 (DE3) strains carrying pET22b-XTR1_285-535_ or pET22b-XYR1_1-195_ were grown at 37℃ and IPTG (isopropyl-β-D-thiogalactopyranoside) was added at a final concentration of 0.5 mM when OD_600_ reached 0.5 ~ 0.6. The recombinant proteins were purified with Ni-nitrilotriacetic acid-agarose (Qiagen, Germany) strictly according to the manufacturer’s instructions.

### Electrophoretic mobility shift assays (EMSAs)

The *xyn1* and *xyn2* promoters were individually divided into several fragments as indicated and each fragment was amplified from the QM9414 genomic DNA. The P*xyn1* (− 321 ~ − 217) fragments carrying indicated site-directed mutagenesis was synthesized in GENEWIZ, Inc. For the EMSA reactions, purified recombinant XTR1 DBD or XYR1 DBD was incubated with 150 ng of the corresponding DNA probe in buffer A (10 mM Tris, 50 mM KCl and 1 mM DTT at pH 7.5) at 20 ℃ for 30 min and subjected to electrophoresis at 4 ℃ using 6% nondenaturing polyacrylamide gels with 0.5 × TBE running buffer and finally stained with ethidium bromide. Competitive EMSAs were similarly performed, except that XTR1 and XYR1 DBDs were simultaneously incubated with the promoter probe P*xyn1* (− 321 ~ − 217).

### DNase I footprinting assay

DNase I footprinting assay was performed as previously described [32]. Briefly, the P*xyn1* (− 321 ~ − 217) fragment was inserted into the pClone007-Blunt-Simple vector to yield pClone007-*xyn1*. With pClone007-*xyn1* as template, PCR reaction was performed using high-fidelity 2X TOLO HIFI DNA Polymerase Premix (TOLO Biotech, Shanghai) and the primer pair M13F/M13R.

Binding reactions were performed with 180 ng of the DNA probe with different amounts of recombinant XTR1-DBD purified from *E.coli* cells. After incubation for 30 min at 25 ℃, 0.015 unit DNase I (Promega, United States) was added to the reaction system and incubation was continued for 1 min at 37 ℃. The digested samples were subjected to phenol extraction to remove protein, followed by ethanol precipitation of DNA. The precipitated DNA was finally dissolved in MILI-Q ultra-pure water and detected by ABI sequencer.

### Sequence analyses

Amino acid sequences of *T. reesei* proteins were retrieved from the JGI databases (https://mycocosm.jgi.doe.gov/Trire2/Trire2.home.html). Sequences of XTR1 orthologues were retrieved from the Uniprot database (https://www.uniprot.org/). Amino acid sequence alignment was performed using ClustalW [35]. Phylogenetic analysis was performed with MEGA7.0 using the Neighbor-joining method with 1000 bootstraps [36].

### Supplementary Information

**Additional file 1: Fig S1.** Self-activation test of the xyn1 or xyn2 promoter fragments when used as baits for yeast one-hybrid assays. Yeast cells carrying pAbAi with promoter fragments including Pxyn1a (-750~-91 bp) (A), Pxyn1b (-1500~-750 bp) (B), Pxyn2a (-750~-43 bp) (C), Pxyn2b (-1500~-750 bp) (D) and Pxyn2 (-1500~-43 bp) (E) were spotted on single dropout medium (-Uracil) with or without AbA supplement, and were allowed to grow at 28°C for 3 days. **Fig S2.** Deletion of xtr1 did not compromise mycelial growth of T. reesei on different carbon sources. (A) Growth of T. reesei QM9414 and Δxtr1 on agar plates at 30°C for 3 days with glucose, lactose, xylose or xylan as the carbon source. (B) Determination of diameters of fungal colonies as indicated in (A). (C) Determination of biomass accumulation in MA medium containing 0.5% (w/v) xylan as carbon source. Error bars are the SD from these replicates. No significant differences (n.s.) were detected in growth between QM9414 and Δxtr1 or CpΔxtr1. **Fig S3.** Deletion of xtr1 hardly affected cellulase production on Avicel cellulose. (A-C) Extracellular cellobiohydrolase (A), ß-glucosidase (B), and endoglucanase (C) activities of the culture supernatant from QM9414 and Δxtr1. (D-F) Transcriptional analyses of cellulase encoding genes including cbh1 (D), bgl1 (E) and eg1 (F) using quantitative RT-PCR. Strains were cultivated on 1% (w/v) Avicel cellulose for the indicated time periods. Values in this figure are the mean of three biological replicates. Error bars are the SD from these replicates. No significant differences were observed in cellulase activities or gene transcription between Δxtr1 and QM9414. **Fig S4.** Analyses of the relative transcriptional level of xtr1 in QM9414, ∆xtr1, and Cp∆xtr1. Strains were cultivated on 0.5% (w/v) xylan for the indicated time periods. **Table S1.** Oligonucleotide primers used in this study.

## Abbreviations

XTR1: Xylanase gene transcriptional repressor 1
ACE1/2/3: Activator of cellulases 1/2/3
CRE1: Carbon catabolite repressor 1
Xpp1: Xylanase promoter-binding protein 1
XYR1: Xylanase regulator 1
SxlR: Specialized xylanase regulator
XYNI: Xylanase I
*xyn1*: Xylanase gene 1
XYNII: Xylanase II
*xyn2*: Xylanase gene 2
SDS-PAGE: Sodium dodecyl sulfate–polyacrylamide gel electrophoresis
Mandels-Andreotti: (MA) medium
SC: Synthetic complete
GFP: Green fluorescence protein
DAPI: 4,,6-Diamidino-2-phenylindole dihydrochloride)
IPTG: Isopropyl-β-D-thiogalactopyranoside
EMSAs: Electrophoretic mobility shift assays
AbA: Aureobasidin A
DBD: DNA binding domain
XAE: Xylanase activating element
qRT-PCR: Quantitative Real-time PCR
