# Supplementary material for: A novel transcriptional repressor specifically regulates xylanase gene 1 in Trichoderma reesei
Source: Biotechnol Biofuels Bioprod. 2023 Oct 27;16:161. doi: 10.1186/s13068-023-02417-w (PMC10612264; doi:10.1186/s13068-023-02417-w)
Supplement: Supplementary file 1 — Additional file 1: Fig S1. Self-activation test of the xyn1 or xyn2 promoter fragments when used as baits for yeast one-hybrid assays. Yeast cells carrying pAbAi with promoter fragments including Pxyn1a (-750~-91 bp) (A), Pxyn1b (-1500~-750 bp) (B), Pxyn2a (-750~-43 bp) (C), Pxyn2b (-1500~-750 bp) (D) and Pxyn2 (-1500~-43 bp) (E) were spotted on single dropout medium (-Uracil) with or without AbA supplement, and were allowed to grow at 28°C for 3 days. Fig S2. Deletion of xtr1 did not compromise mycelial growth of T. reesei on different carbon sources. (A) Growth of T. reesei QM9414 and Δxtr1 on agar plates at 30°C for 3 days with glucose, lactose, xylose or xylan as the carbon source. (B) Determination of diameters of fungal colonies as indicated in (A). (C) Determination of biomass accumulation in MA medium containing 0.5% (w/v) xylan as carbon source. Error bars are the SD from these replicates. No significant differences (n.s.) were detected in growth between QM9414 and Δxtr1 or CpΔxtr1. Fig S3. Deletion of xtr1 hardly affected cellulase production on Avicel cellulose. (A-C) Extracellular cellobiohydrolase (A), ß-glucosidase (B), and endoglucanase (C) activities of the culture supernatant from QM9414 and Δxtr1. (D-F) Transcriptional analyses of cellulase encoding genes including cbh1 (D), bgl1 (E) and eg1 (F) using quantitative RT-PCR. Strains were cultivated on 1% (w/v) Avicel cellulose for the indicated time periods. Values in this figure are the mean of three biological replicates. Error bars are the SD from these replicates. No significant differences were observed in cellulase activities or gene transcription between Δxtr1 and QM9414. Fig S4. Analyses of the relative transcriptional level of xtr1 in QM9414, ∆xtr1, and Cp∆xtr1. Strains were cultivated on 0.5% (w/v) xylan for the indicated time periods. Table S1. Oligonucleotide primers used in this study. [file 13068_2023_2417_MOESM1_ESM.docx]

**A novel transcriptional repressor specifically regulates xylanase gene 1 in *Trichoderma reesei***

Wenqiang Xu^1, 2,^ ^#^, Yajing Ren^1, #^, Yuxiao Xia^1^, Lin Liu^1^, Xiangfeng Meng^1^, Guanjun Chen^1^, Weixin Zhang^1,^*, Weifeng Liu^1^

^1^State Key Laboratory of Microbial Technology, Shandong University, No.72 Binhai Road, Qingdao 266237, P. R. China

^2^ Shandong Lishan Biotechnology Co., LTD

^#^These authors contribute equally.

^*^Correspondence should be addressed to W Zhang. E-mail: zhangwx@sdu.edu.cn

**Additional file Information**


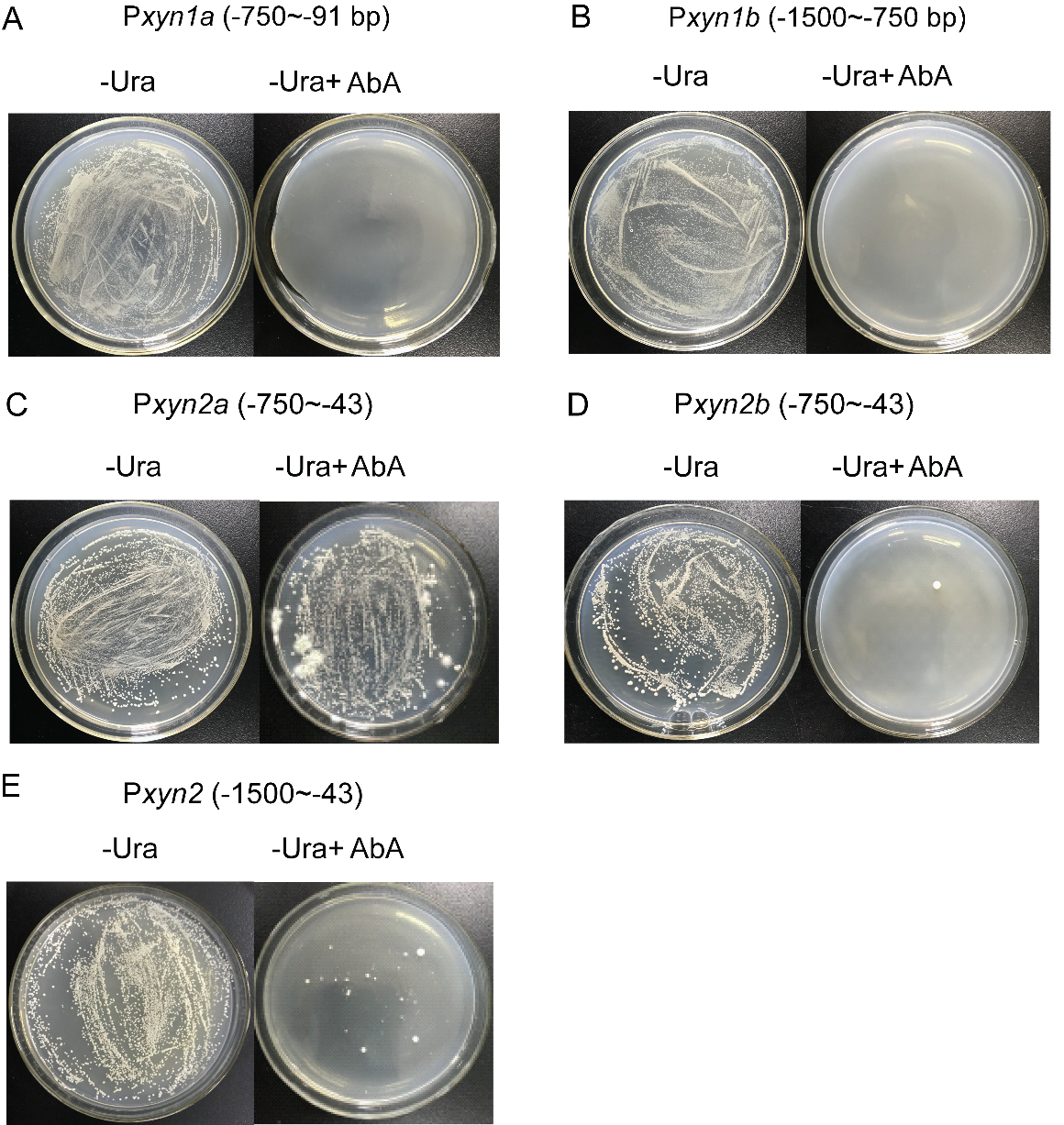


**Fig. S1 Self-activation test of the *xyn1* or *xyn2* promoter fragments when used as baits for yeast one-hybrid assays.** Yeast cells carrying pAbAi with promoter fragments including P*xyn1a* (-750~-91 bp) (A), P*xyn1b* (-1500~-750 bp) (B), P*xyn2a* (-750~-43 bp) (C), P*xyn2b* (-1500~-750 bp) (D) and P*xyn2* (-1500~-43 bp) (E) were spotted on single dropout medium (-Uracil) with or without AbA supplement, and were allowed to grow at 28°C for 3 days.


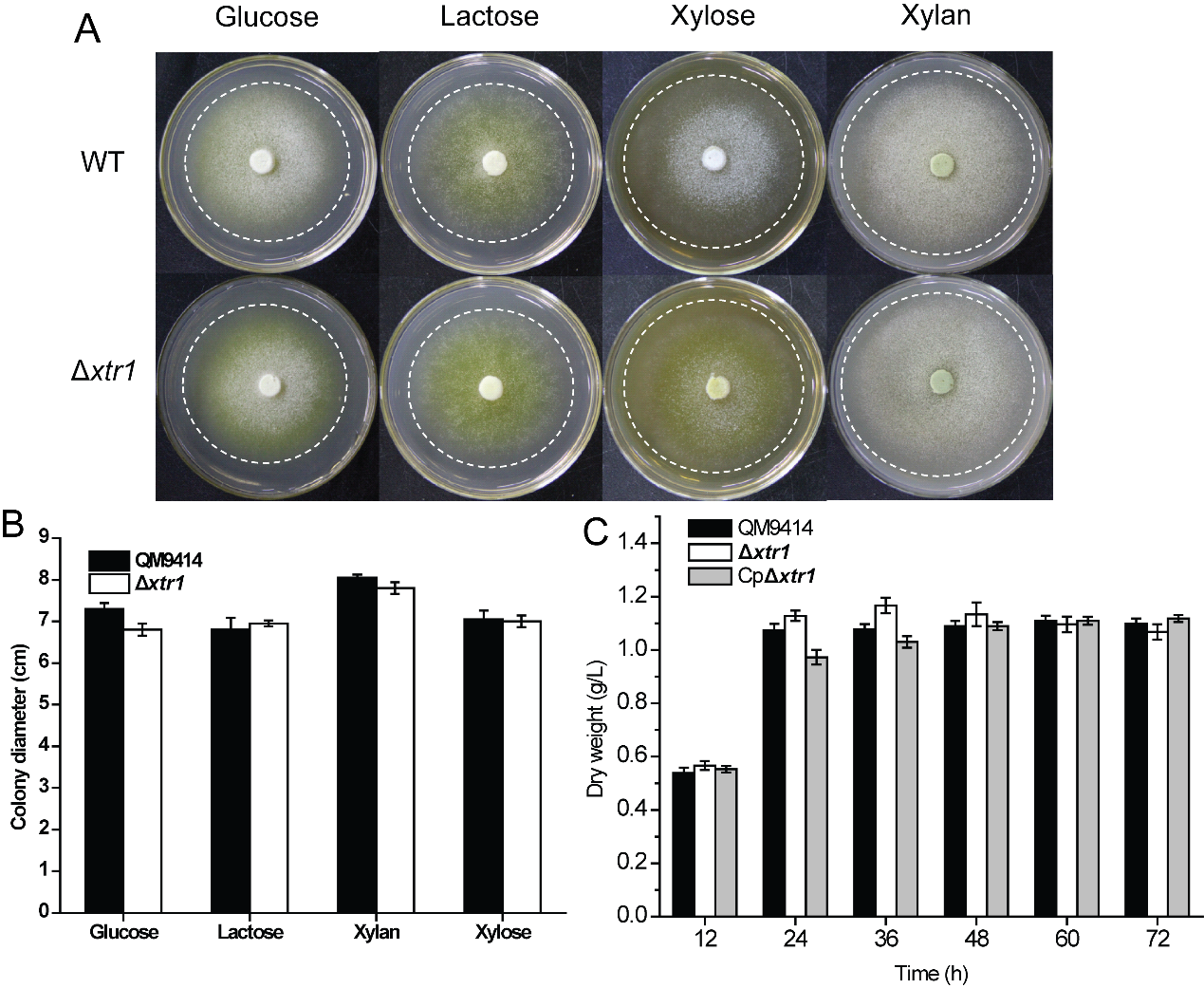


**Fig. S2 Deletion of *xtr1* did not compromise mycelial growth of *T. reesei* on different carbon sources.** (A) Growth of *T. reesei* QM9414 and Δ*xtr1* on agar plates at 30°C for 3 days with glucose, lactose, xylose or xylan as the carbon source. (B) Determination of diameters of fungal colonies as indicated in (A). (C) Determination of biomass accumulation in MA medium containing 0.5% (w/v) xylan as carbon source. Error bars are the SD from these replicates. No significant differences (n.s.) were detected in growth between QM9414 and Δ*xtr1* or CpΔ*xtr1*.

**
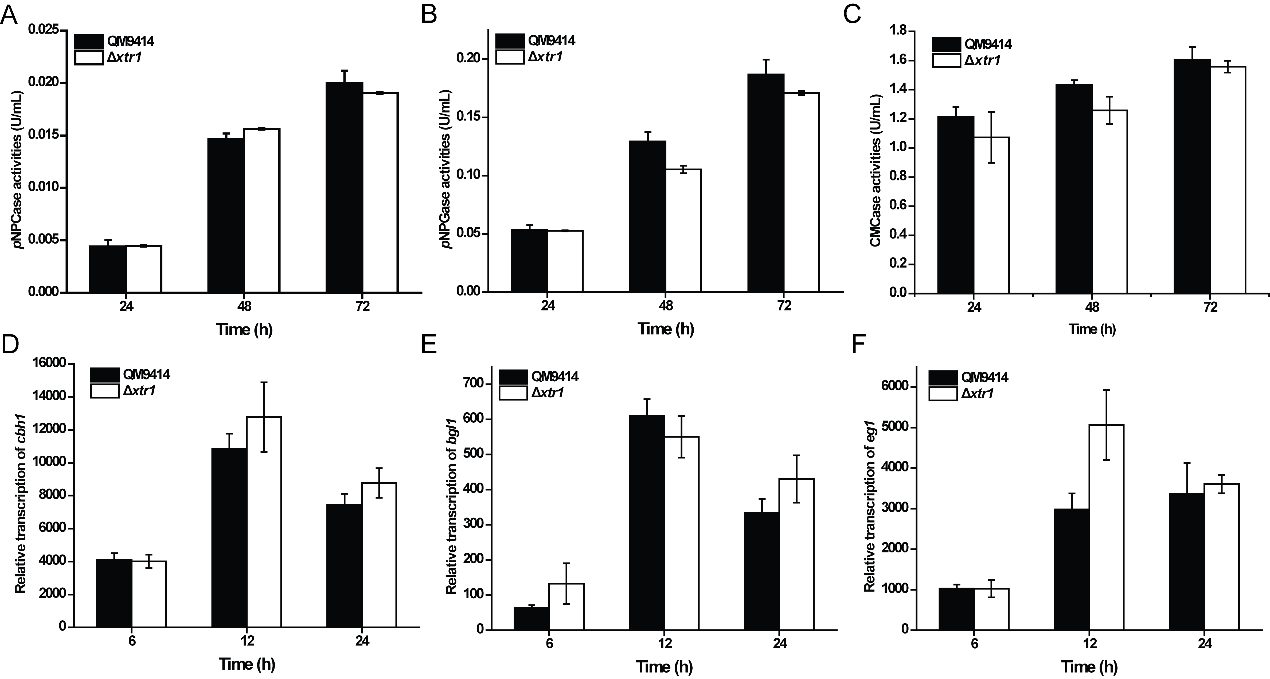
**

**Fig. S3 Deletion of *xtr1* hardly affected cellulase production on Avicel cellulose.** (A-C) Extracellular cellobiohydrolase (A), ß-glucosidase (B), and endoglucanase (C) activities of the culture supernatant from QM9414 and Δ*xtr1*. (D-F) Transcriptional analyses of cellulase encoding genes including *cbh1* (D), *bgl1* (E) and *eg1* (F) using quantitative RT-PCR. Strains were cultivated on 1% (w/v) Avicel cellulose for the indicated time periods. Values in this figure are the mean of three biological replicates. Error bars are the SD from these replicates. No significant differences were observed in cellulase activities or gene transcription between Δ*xtr1* and QM9414.

**
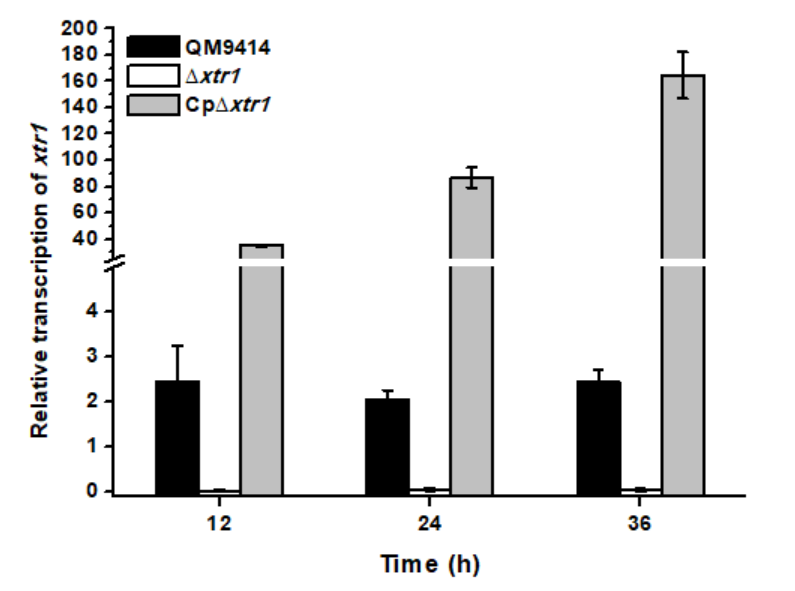
**

**Fig. S4 Analyses of the relative transcriptional level of *xtr1* in QM9414,** **∆*xtr1*, and Cp∆*xtr1*.** Strains were cultivated on 0.5% (w/v) xylan for the indicated time periods.

**Table S1 Oligonucleotide primers used in this study.**

| **Primer** | **Sequence (5'-3')** | **Application** |
| --- | --- | --- |
| F*60132*-up | TAGGGATAACAGGGTAATGGAAAGGGGATGAGGTGAAGG | Used for plasmid construction for Tr*60132* (*xtr1*) deletion |
| R*60132*-up | GGGGACAAGTTTGTACAAAAAAGCAGGCTAAGCTGGTCCATGCGAATAGAG |  |
| F*60132*-down | GGGGACCACTTTGTACAAGAAAGCTGGGTAGGATGGATGGAAGCCAAACCT |  |
| R*60132*-down | ATTACCCTGTTATCCCTACTCACAACATTCACCACCACAGC |  |
| Fre*60132* | ACGACCTGGTTGATACGACAATGCCCAGTGGCCCCGGCTC | Primers used for complementation of ΔTr*60132* |
| Rre*60132* | CGGGATCTTGCAGGCCGGGCGTGAGGTTGGAAGGCTGGATTC |  |
| Fabai-*xyn1*(-1500 to -750) | GAATTCGAGCTCGGTACCCGGGCTTGGGATAGGCCAGACC | Primers for the construction of plasmids used in yeast one-hybrid assays |
| Rabai-*xyn1*(-1500 to -750) | GAGCACATGCCTCGAGGTCGACCTAGAGCTCCTGTAAGTCT |  |
| Fabai-*xyn1*(-750 to -91) | GAATTCGAGCTCGGTACCCGGGAGACTTACAGGAGCTCTAG |  |
| Rabai-*xyn1*(-750 to -91) | GAGCACATGCCTCGAGGTCGACTATGCAGAATTCTTCCAC |  |
| Fabai-*xyn2*(-1500 to -750) | GAATTCGAGCTCGGTACCCGGGGATCATAGACGCAGCCAGC |  |
| Rabai-*xyn2*(-1500 to -750) | GAGCACATGCCTCGAGGTCGACCATTGGTCTAGTGCCGAT |  |
| Fabai-*xyn2*(-750 to -43) | GAATTCGAGCTCGGTACCCGGGATCGGCACTAGACCAATGG |  |
| Rabai-*xyn2*(-750 to -43) | GAGCACATGCCTCGAGGTCGACAGACAGTTGACGGATAATTTG |  |
| F-his-*xtr1* | TTTAAGAAGGAGATATACATATGGGGGGCCGGAATGGTTAC | Primers used for heterologous expression of XTR1 DBD |
| R-his-*xtr1* | GTGGTGGTGGTGGTGGTGCTCGAGTGAGGTTGGAAGGCTGG |  |
| F-his-*xyr1* | TTTAAGAAGGAGATATACATATGATGTTGTCCAATCCTCTCCGTC |  |
| R-his-*xyr1* | GTGGTGGTGGTGGTGGTGCTCGAGCGCTTTGCTGGCGTGAGAGTTTGC |  |
| F-emsa-*xyn2*(-1500 to -750) | GATCATAGACGCAGCCAGC | Primers used for amplification of DNA fragment for EMSAs |
| R-emsa-*xyn2*(-1500 to -750) | CCATTGGTCTAGTGCCGAT |  |
| F-emsa-*xyn2*(-750 to -43) | ATCGGCACTAGACCAATGG |  |
| R-emsa-*xyn2*(-750 to -43) | AGACAGTTGACGGATAATTTG |  |
| F-emsa-*xyn1*(-1500 to -750) | CTTGGGATAGGCCAGACC |  |
| R-emsa-*xyn1*(-1500 to -750) | CTAGAGCTCCTGTAAGTCT |  |
| F-emsa-*xyn1*(-750 to -91) | AGACTTACAGGAGCTCTAG |  |
| R-emsa-*xyn1*(-750 to -91) | TATGCAGAATTCTTCCAC |  |
| R-emsa-*xyn1*(-750 to -538) | TGCTGCAGAATTGTAGGACT |  |
| F-emsa-*xyn1*(-538 to -321) | AACTAGGACCGGCTTGTG |  |
| R-emsa-*xyn1*(-538 to -321) | TATGCAGAATTCTTCCAC |  |
| F-emsa-*xyn1*(-321 to -91) | AACTAGGACCGGCTTGTG |  |
| R-emsa-*xyn1*(-321 to -217) | GGCATTTTGCCTAAAGCACG |  |
| F-emsa-*xyn1*(-217 to -91) | AAGCTTCGAGGATACTGTAC |  |
| F-emsa-mutant1 | GTTTGATCCGATACCTAA |  |
| F-emsa-mutant2 | GTTTGATCCGATACCTGGAAAA |  |
| F-emsa-mutant3 | AGGGAAGAATTTAGGCAAAATGC |  |
| F-emsa-mutant4 | ATACCTGGAGGGCGTGCGGG |  |
| qF*60132* | TGAAGACGAGAAGTGCATGC | Primers used for quantitative RT-PCR analyses |
| qR*60132* | TGAAGACGAGAAGTGCATGC |  |
| qF*cbh1* | CTTGGCAACGAGTTCTCTT |  |
| qR*cbh1* | TGTTGGTGGGATACTTGCT |  |
| qF*eg1* | CGGCTACAAAAGCTACTACG |  |
| qR*eg1* | CTGGTACTTGCGGGTGAT |  |
| qF*bgl1* | AGTGACAGCTTCAGCGAG |  |
| qR*bgl1* | GGAGAGGCGTGAGTAGTTG |  |
| qF*xyn1* | AAACTACCAAACTGGCGG |  |
| qR*xyn1* | TTGATGGGAGCAGAAGATCC |  |
| qF*xyn2* | CGGCTACTTCTACTCGTACTG |  |
| qR*xyn2* | TTGATGACCTTGTTCTTGGTG |  |
| qF*actin* | TGAGAGCGGTGGTATCCACG |  |
| qR*actin* | GGTACCACCAGACATGACAATGTTG |  |
